# Effects of Plant Size, Temperature, and Light Intensity on Flowering of *Phalaenopsis* Hybrids in Mediterranean Greenhouses

**DOI:** 10.1155/2014/420807

**Published:** 2014-11-20

**Authors:** Roberta Paradiso, Stefania De Pascale

**Affiliations:** Department of Agricultural Sciences, University of Naples Federico II, Via Università 100, 80055 Portici, Naples, Italy

## Abstract

Mediterranean greenhouses for cultivation of* Phalaenopsis* orchids reproduce the warm, humid, and shaded environment of tropical underbrush. Heating represents the highest production cost, due to the high thermal requirements and the long unproductive phase of juvenility, in which plants attain the critical size for flowering. Our researches aimed to investigate the effect of plant size, temperature, and light intensity, during the phase of flower induction, on flowering of modern genotypes selected for Mediterranean greenhouses. Three experiments were carried out to compare (i) plant size: reduced size versus size considered optimal for flowering (hybrids “Sogo Yukidian,” “Chain Xen Diamond,” and “Pinlong”); (ii) temperature: moderate reduction of temperature versus standard thermal regime (hybrid “Premium”); (iii) light intensity: supplemental lighting versus reference light intensity (hybrid “Premium”). The premature exposure of plants to the inductive treatment delayed the beginning of flowering and reduced the flower stem quality, in all the tested hybrids. In “Premium,” the lower temperature did not affect flowering earliness and commercial quality of flower stems compared to the standard regime, whereas it promoted stem branching. In the same hybrid, supplemental lighting anticipated flowering and promoted the emission of the second stem and the stem branching, compared to the reference light regime.

## 1. Introduction

Commercial production of* Phalaenopsis* orchids increased considerably worldwide in the last two decades [1].

*Phalaenopsis* is a genus of tropical orchids exhibiting crassulacean acid metabolism (CAM) [2]. In the native* habitat*, temperatures range throughout the year from 28 to 35°C in the day and from 20 to 24°C in the night, and plants act as epiphytic, growing on tree trunks and limbs, shaded by the dense canopy of the forest [3].

Greenhouses in Mediterranean climate reproduce the natural warm and humid* habitat*, so that air and basal heating are used for most of the time of the year [4, 5].

**Figure 1:**
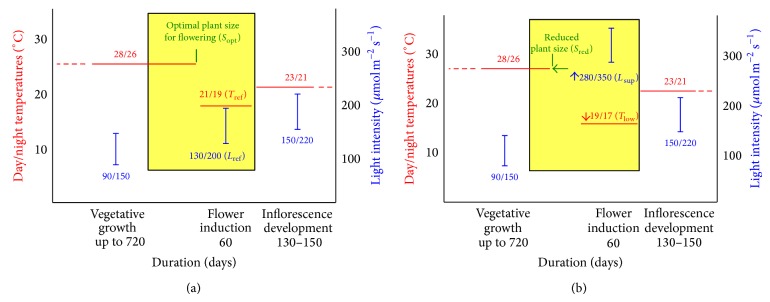
(a) References for duration, day/night temperatures, and light intensity for the different phases of cultivation of* Phalaenopsis* orchids in Mediterranean greenhouses. (b) Proposed changes for the optimization of the cultivation protocol. Reduced plant size (*S*_red_) corresponds to 2 leaves fewer than the optimal sizes (*S*_opt_); moderately lower regime (*T*_low_) corresponds to a 2°C decrease compared to the standard thermal regime (*T*_ref_); supplemental lighting (*L*_sup⁡_) corresponds to a constant additional PPFD of 150 *μ*mol m^−2^ s^−1^ for 6 hours per day compared to the reference light regime (*L*_ref_).

Similarly to other orchids,* Phalaenopsis* shows a juvenile phase, in which plants must reach a certain stage of growth to attain the capacity to flower [6]. Temperature is the primary environmental factor to initiate flowering [7, 8]. In greenhouse production in Italy, plants are commonly grown at day/night (D/N) temperatures of 28/26°C (up to 24 months, depending on the hybrid), to promote growth until the critical size for flowering (*vegetative phase*). Afterwards, plants are stimulated to flower through a “cooling” treatment at 21/19°C (30 to 60 days,* flower induction phase*) and finally exposed to 23/21°C (100 to 150 days) to hasten the inflorescence development (*finishing phase*) (Figure 1). For this last phase, the shorter time is required for potted plants, sold after the first flower anthesis, and the longer time for cut stems, harvested at the complete anthesis of the inflorescence.

The juvenile phase of* Phalaenopsis* is relatively long and greatly varies among varieties and hybrids, ranging from 6 to over 24 months from the transplanting of young micropropagated plants. Consistent with this variability, the growth stage considered as optimal to expose plants to the thermal treatment for flower induction also varies from 5 to 7 fully expanded leaves (with a leaf length from 15 to 25 cm) [4, 5], even though information on the effect of plant size on flowering characteristics is still scarce for current genotypes.

Temperature constantly higher than 26°C promotes the vegetative growth and inhibits flower transition in* Phalaenopsis*, while reduction of temperatures below 26°C, especially during the day, can induce flowering even in immature plants [4]. However, the early flower induction by cool nights during the vegetative phase can be prevented by keeping the day temperature sufficiently high [9]. Data on the effects of the level of inductive temperatures on flowering earliness and characteristics are limited [9, 10]. In some old selections, decreasing temperatures from 25 to 14°C delay flowering and reduce the percentage of flowering plants, but they increase the number of flowers per inflorescence [9]. For modern varieties and hybrids with very different genetic backgrounds (and presumably different thermal requirements for growth and flowering), the specific sensitivity to temperature is not well known; however it seems to vary greatly [11].

Light requirement of* Phalaenopsis* is relatively low, due to the plant adaptation to the dim light of tropical underbrush [3]. The threshold of light tolerance changes throughout the plant developmental stages, increasing from juvenility to maturity (Figure 1). Accordingly, shading of the greenhouse compartments at different limits of photosynthetic photon flux density (PPFD) is extensively adopted by growers [4, 12]; however the effect of light intensity on the process of flowering is not clear. In fact, higher light intensities before [13–15] or during [16] the inductive treatment promote flowering in some old hybrids, but no data seems to be available on modern genotypes selected for Mediterranean greenhouses.

Due to the long duration of the growing cycle and the high thermal requirements, particularly for the vegetative growth, greenhouse cultivation of* Phalaenopsis* is very expensive, and heating is one of the highest costs in Mediterranean areas, where this species is nowadays largely produced [11]. In this respect, knowledge of the real critical plant size for flowering in the most common among the numerous genotypes would permit anticipating the flower induction, while shortening the long unproductive vegetative phase. Besides, a better understanding of thermal and light requirements during flowering could allow optimizing the strategies for temperature and light control in greenhouse, while improving the production process.

On this basis, our researches aimed to investigate the effect of moderate variations of the reference plant size, D/N temperatures, and light intensity, in the phase of flower induction, on the process of flowering and the final quality of cut flower stems and flowering potted plants of* Phalaenopsis*.

## 2. Materials and Methods

A series of 3 experiments was carried out in heated glasshouse to assess the effects on flowering time and flower stem production and characteristics of the following treatments:

- plant size: reduced size (2 leaves fewer) compared to the size considered optimal for flowering (3 hybrids with different morphology);
- temperature: 2°C decrease with respect to the standard thermal regime (hybrid “Premium”);
- light intensity: supplemental lighting compared to the reference light intensity (hybrid “Premium”).

### 2.1. Glasshouse and Growth Conditions

The experiments were carried out in Naples (40°51′N, 14°22′E), in a commercial glasshouse, including separate compartments for each phenological phase: vegetative growth, flower induction, and inflorescence development (Figure 1).

Plants were grown in 12 cm transparent plastic pots, on a mixture of bark (95%) and sphagnum (5%), on mobile benches, at a density of 48 plants/m^2^. The cultivation protocol commonly used in the commercial practice was adopted.

In all the three experiments, plants from the vegetative compartment were exposed to the thermal treatment for flower induction (normally 21/19°C, for 60 days) and then moved to the finishing phase (23/21°C), until the complete anthesis of the inflorescence (Figure 1).

The compartment for flower induction was heated via basal heating (hot water system) and shaded to keep PPFD at the canopy level below 200 *μ*mol m^−2^ s^−1^. Shading consisted in plastic films and black nets, with a shading rate ranging from 20% to 70%. The high temperature and low relative humidity (RH) in spring and summer were controlled by a cooling system (RH set point 70%) and by further shading the greenhouse, whitewashing the roof starting from April.

Plants were fertigated via a drip system (1 per pot; 2 L/h), with reverse osmosis water (electrical conductivity, EC = 70 *μ*S/cm at 25°C). The N : P : K ratio in the nutrient solution was 1 : 0.5 : 1; pH and EC were kept at 6.5 and 1200 *μ*S/cm, respectively [7, 8]. Each pulse lasted 3 minutes and the number of pulses varied from one every 5 days (from the end of December to the end of February) to one every 3 days (from the beginning of March to the end of July). Fertigation was alternated with one irrigation every 5 pulses, to prevent the salt accumulation in the substrate.

### 2.2. Experimental Treatments

**Experiment 1 (effects of plant size).**

The experiment was carried out from December to June.

Three hybrids of* Phalaenopsis* with different morphological characteristics were selected among those suggested for cultivation in Mediterranean greenhouses: “Sogo Yukidian” (Large), “Chain Xen Diamond” (Medium), and “Pinlong” (Small) (Table 1).

The flower induction treatment was performed from December 9 to February 7.

The following plant size at the moment of flower induction was compared:

- the optimal size (*S*_opt_), corresponding to 5 leaves per plant in the Large and Small hybrids, and 7 leaves per plant in the Medium hybrid;
- a reduced size (*S*_red_), corresponding to 2 leaves fewer than the optimal sizes (3 and 5 leaves per plant, resp.).

The average age of the plants in the different hybrids was approximately: 30 weeks (210 days) in Large, 38 weeks (266 days) in Medium, 25 weeks (175 days) in Small, for the *S*_opt_, and 18 weeks (126 days), 28 weeks (196 days), 15 weeks (105 days), respectively, in the *S*_red_.

**Experiment 2 (effects of thermal regime).**

The experiment was carried out from December to July, on 2-year plants of* Phalaenopsis* “Premium” (white flower), one of the most common hybrids in Europe for both cut stems and potted plants production.

Two D/N thermal regimes were compared during the phase of flower induction, from December 28 to February 28:

- 21/19°C, the reference regime commonly adopted in commercial farms (*T*_ref_);
- 19/17°C, a moderately lower regime (*T*_low_).

The inductive treatments were applied on 2-year plants at the growth stage considered optimal for flowering (7 leaves per plant, corresponding to a leaf area of approximately 580 cm^2^).

Actual average values of air temperature under the two inductive regimes were 21.1/18.4 (*T*_ref_) and 19.5/16.3 (*T*_low_).

**Experiment 3 (effects of light intensity).**

The experiment was carried out from January to August, on 2-year plants of* Phalaenopsis* “Premium.”

Two lighting regimes were compared during the inductive treatment, from January 13 to March 13:

- the reference light regime (*L*_ref_), obtained by shading the canopy at the PPFD threshold of 200 *μ*mol m^−2^ s^−1^;
- supplemental lighting (*L*_sup⁡_), provided by 400 W HPS lamps, placed at 120 cm from the bench, to obtain a constant additional PPFD to the reference light of 150 *μ*mol m^−2^ s^−1^, at the plant level. Lighting treatment lasted 6 hours per day (3 hours in the morning and 3 hours in the afternoon), within the natural day length of the period.

### 2.3. Measurements and Data Handling

Temperature and R.H. in the greenhouse were measured hourly with data loggers Tinytag Ultra 2 (Gemini Data Loggers Ltd., Chichester, West Sussex, UK), placed on the benches. Light intensity at the canopy level was recorded hourly by using a Delta OHM multifunction meter DO-9847 (Delta Ohm, Padova, Italy).

At the beginning and at the end of the experiments, 5 plants per treatment were collected to measure the number of leaves, the individual leaf area, and the total leaf area, with a LICOR 3000 area meter.

Each experiment was carried out on 25 plants per treatment.

**Figure 2:**
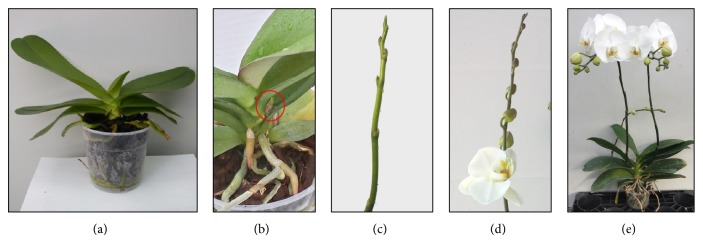
Developmental stages of* Phalaenopsis*: emergence of flower stem ((a), (b)), appearance of visible flower buds (c), and anthesis of the first flower (d) and of the inflorescence (e).

Flowering time was determined on 25 plants per treatment as days from the beginning of the inductive treatment (DBT) to the emergence of flower stem, the first flower anthesis (beginning of flowering, corresponding to the commercial maturity for potted plants), and the complete anthesis of the inflorescence (commercial maturity for cut stems) (Figure 2).

At the complete anthesis, the characteristics of the main flower stem (stem and inflorescence length, stem diameter, number of flowers) were measured on 10 plants per treatment. Stem diameter was measured by using a digital caliper (Mitutoyo Ltd, UK). The number of stems with branches and the number of plants with two stems were determined as percentage of the total number of plants per treatment.

Data were analysed by ANOVA and means were compared by the least significant differences (LSD) test, at *P* = 0.05.

## 3. Results

### 3.1. Effects of Plant Size

**Figure 3:**
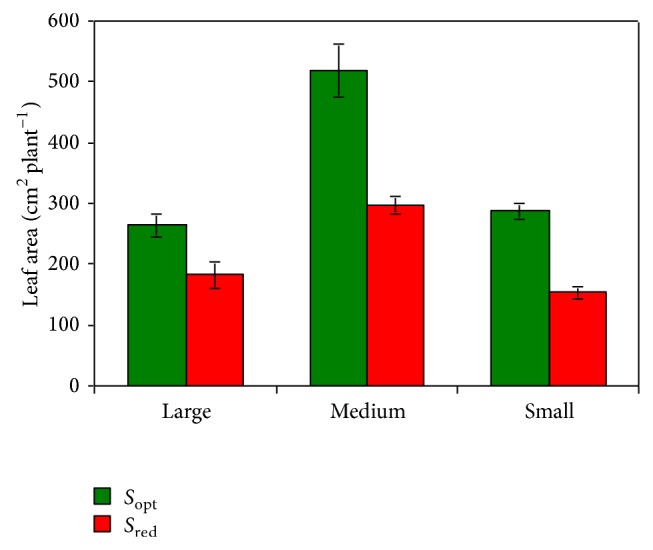
Total plant leaf area at the flower induction in the three* Phalaenopsis* hybrids (Large, Medium, and Small), as a function of the plant size (mean value ± standard error; *n* = 5). Experiment 1 (effects of plant size): *S*_opt_ = 5 leaves per plant in the Large and Small hybrids, and 7 leaves per plant in the Medium hybrid; *S*_red_ = 3 and 5 leaves per plant, respectively.

The leaf area of plants at the size considered optimal for flowering was similar in the Large and Small hybrids (275 cm^2^ per plant on average) while it was greater in the Medium one (517 cm^2^ per plant) (Figure 3). This was due to both the higher number of leaves (7 versus 5 leaves per plant) and the larger individual leaf area (74 cm^2^ in Medium versus 55 cm^2^ per leaf on the average in the other two hybrids). Plants with reduced size (2 leaves fewer) showed different decreases in the total leaf area compared to those at the optimal size (−31% in Large, −42% in Medium, and −47% in Small).

The flowering process was influenced by both the hybrid and the plant size at the moment of the inductive treatment. The emergence of flower stem started earlier in Medium and Large plants and later in the Small ones, and it was significantly delayed in the reduced size compared to the optimal size (Table 2). Accordingly, the anthesis of the first flower occurred earlier in the Medium and Large than in the Small hybrid, and it was always delayed in younger plants (Table 2).

The flower stem characteristics varied in the hybrids, as expected (Table 3). The Small plants showed the shortest stem and inflorescence but the highest number of flowers. The plant size significantly influenced all the quality parameters (Table 3). The reduced size always determined shorter stem and inflorescence and fewer flowers (Table 3). The negative effect of the early flower induction on stem quality was lighter in the Large hybrid (Data not shown).

Branching of flower stem depended on the hybrid, occurring only in the Small one, and was unaffected by the plant size (83% of the total number of plants, on average). The 100% of the plants in all the three hybrids produced the second flower stem, regardless of the plant size.

### 3.2. Effects of Thermal Regime

In plants of* Phalaenopsis* “Premium,” the number of leaves did not change significantly during the 60-day inductive treatment and was not affected by the thermal regime (7.0 leaves per plant on average). At the end of this phase, individual leaf area and total plant leaf area were also similar in the two regimes (94.9 cm^2^ per leaf and 664.3 cm^2^ per plant on average, resp.). Further details on the effects of thermal regime on plant growth in this experiment are reported in Paradiso et al., 2012 [17].

Under the reference regime, the emergence of flower stem started at 47 DBT on average (Table 2) and was completed in all the plants at 66 DBT (6 days after the end of the inductive treatment). Flowering began at 161 DBT with the anthesis of the first flower and was completed in 196 days, with the complete anthesis of the inflorescence (Table 2). The stem emergence was slightly late (55 DBT on average) (Table 2) and slower (84 DBT to be completed) under the lower temperatures, so that the stem was present in only 64% of the plants at the end of the inductive treatment. However, all the plants developed the inflorescence even under the *T*_low_ regime, which did not affected significantly the time for the anthesis of the first flower and the complete anthesis of the inflorescence compared to the *T*_ref_ (Table 2).

Flower stems in plants induced under *T*_ref_ reached the length of 66 cm and formed inflorescences 27 cm long, with 10 flowers (Table 3). Under this regime, the 29% of the stems had lateral branches at the first node below the inflorescence and the 25% of the plants formed the second flower stem. *T*_low_ reduced the length of the stem, mainly by shortening the inflorescence (−5 cm), while it promoted the emission of lateral branches and of the second flower stem, which appeared in 100% and 36% of the plants, respectively.

### 3.3. Effects of Light Intensity

**Figure 4:**
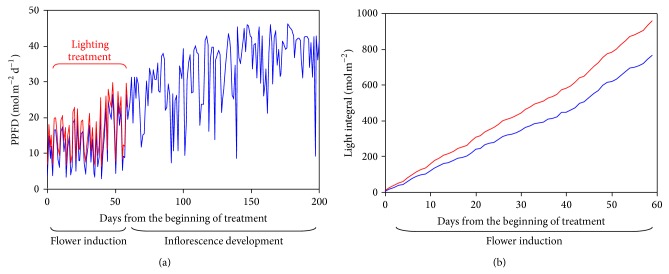
(a) Daily values of natural light intensity recorded during the experiment in the greenhouse compartments for flower induction and inflorescence development in the reference light regime (*L*_ref_, blue line) and under supplemental lighting (*L*_sup⁡_, red line). (b) Light integral recorded during the phase of flower induction in *L*_ref_ (blue line) and *L*_sup⁡_ (red line).  Experiment 3 (effects of light intensity): supplemental lighting (*L*_sup⁡_) corresponds to a constant additional PPFD of 150 *μ*mol m^−2^ s^−1^ for 6 hours per day compared to the reference light regime (*L*_ref_).

Under the reference lighting regime, the daily values of the light intensity recorded in the compartments for flower induction (January 13–March 13) and inflorescence development (March 14–July 31) varied from 2.98 mol m^−2^ d^−1^, in some cloudy days in the winter, to 46.17 mol m^−2^ d^−1^, in several days from the beginning of June (Figure 4(a)).

The light integral determined at the canopy level at the end of the 60-day inductive phase in *L*_ref_ was 763 mol m^−2^ (Figure 4(b)). Supplemental lighting resulted in 3.24 mol m^−2^ of additional PPFD per day and increased the light integral to 957 mol m^−2^ (Figure 4(b)).

In plants of* Phalaenopsis* “Premium,” the number of leaves did not change during the inductive period and it was not affected by lighting conditions (7.7 leaves per plant, on average). Similarly, the individual leaf area and total plant leaf area at the end of the treatment were similar under the two lighting regimes (99.0 cm^2^ per leaf and 693.6 cm^2^ per plant, resp.).

Under *L*_ref_, the emergence of the flower stem started at 43 DBT on average (Table 2), and it was completed in all the plants at 51 DBT. The anthesis of the first flower and of the entire inflorescence occurred in 149 and 185 DBT, respectively (Table 2). Under *L*_sup⁡_, the stem emergence was anticipated (−7 days to start and −10 days to be completed), and the flowering was slightly early (−6 and −5 days for the beginning and the complete anthesis) compared to *L*_ref_ (Table 2).

The light integral recorded at the canopy level at the first flower anthesis was 3490 mol m^−2^ on average, with very close values in *L*_ref_ and *L*_sup⁡_ (data not shown).

The light intensity during the inductive treatment did not affect significantly the characteristics of the main flower stem while it influenced the plant architecture (Table 3). In fact, supplemental lighting promoted the emission of the second flower stem (from 40% in *L*_ref_ to 75% in *L*_sup⁡_ of the plants) and the formation of lateral branches in both the flower stems (from 50% in *L*_ref_ to 75% in *L*_sup⁡_). Accordingly, stems obtained under *L*_sup⁡_ showed higher values of dry matter compared to *L*_ref_ (25.0 versus 23.6 g per stem).

## 4. Discussion

### 4.1. Effects of Plant Size

Plants of the three hybrids of* Phalaenopsis* showed different responses to the inductive stimuli, with the appearance of the flower stems in the third (Medium), the fourth (Large), and the fifth week (Small) after the beginning of the treatment. These reaction times place these hybrids among the earliest modern genotypes [5] and they are shorter than those of other hybrids commonly grown in Europe, such as “Premium.” This result suggests that the duration of the inductive phase could be adjusted to the different genotypes, and shorter times of exposure to cooling and earlier transfer to the finishing compartment could be applied to faster hybrids.

Compared to the optimal size, the smaller plant size at the moment of flower induction influenced the process of flowering in all the tested hybrids, slightly delaying stem emergence and flower anthesis, and impairing the stem characteristics. These effects confirm that an appropriate plant size/age is crucial in* Phalaenopsis* to react to inductive temperatures and to sustain the high energetic demand for inflorescence development and seed production [18]. Nevertheless, thermal stimulation of less mature plants did not impede flower transition at the sizes/ages considered in our experiment (2 fewer leaves than optimum, corresponding to 10 to 12 weeks younger age), proving that even smaller plants had attained the competence to flower [5].

The plant size influenced the time for stem emergence more than the time for flowering. Indeed, once the stem was emerged, the number of days for flower anthesis was unaffected by the plant size. In addition, the response to the early induction depended on the hybrid, highlighting that the genotype-specific sensitivity to this practice should be investigated, in order to adapt the cultivation protocol to the plant material. Indeed, since in the most common hybrids a new leaf emerges every six weeks on average, anticipating the inductive treatment as tested in our experiment would reduce, by approximately 3 months, the phase of vegetative growth, which is the longest and most expensive in greenhouse production of* Phalaenopsis*.

### 4.2. Effects of Thermal Regime

In the 21/19°C reference regime, flower stems of* Phalaenopsis* “Premium” started to appear during the sixth week after the beginning of the inductive treatment, and they were present in all the plants by the middle of the tenth week. This reaction time is longer than those observed in the hybrids of Experiment 1 and than those obtained in 2-year plantsof other modern hybrids, in which three to four weeks of cooling were sufficient to obtain visible stems [9]. It is important to note that sensitivity to inductive temperature in* Phalaenopsis*, in terms of both time of exposure needed and time of reaction, not only depends on the plant age and size, but is also influenced by the “thermal past” (duration and level of temperature in the vegetative period) [9] and by the plant genetic background [5, 19, 20].

Under the reference temperatures, the production cycle lasted approximately 23 weeks (first flower anthesis) and 28 weeks (entire inflorescence anthesis), for flowering potted plants and cut stem production, respectively, confirming “Premium” as a late flowering hybrid [9, 21].

The 2-degree decrease of the inductive temperatures did not affect the time for flowering. It is likely that, under our experimental conditions, this reduction was not sufficient to determine a significant effect in the rate of stem elongation and inflorescence development, as expected [22]. Our result contradicts previous studies which proved that 17–19°C is lower than the optimal temperatures (24–26/17–19°C for 45 days) to induce flowering in this hybrid (also called “V3” in China) [23, 24].

Lower temperatures reduced the length of stem and inflorescence and the number of flowers, which are considered the main parameters for cut stem grading. However, this did not influence the overall commercial quality, since stems were much longer than the minimum required for the first grade in this type of hybrids (40 cm) [25]. In addition, as observed in old oriental hybrids [26], lower temperatures promoted stem branching, which is among the main factors for price determination of potted orchids. This prefigures that even in* Phalaenopsis*, the inductive regime could be changed depending on the production purpose, since compact shape and branched stems are positively evaluated in flowering potted plants.

### 4.3. Effects of Light Intensity

Flowering of* Phalaenopsis* “Premium” was positively influenced by supplemental lighting during the inductive phase, which reduced from 51 to 41 days the time to obtain the stem emergence in all the plants. It also anticipated the first flower anthesis by 6 days, compared to the reference light. Similarly, Higuchi et al. [16] found that light intensity higher than 300 *μ*mol m^−2^ s^−1^ during flower induction promoted flowering in old Asiatic hybrids. In the same hybrids, Inoue and Higuchi [27, 28] suggested that 96–190 *μ*mol m^−2^ s^−1^ of artificial light was suitable for practical purposes, in the experimental conditions adopted in their experiment.

Our result is relevant for production scheduling of “Premium” that is a slow reactive hybrid. Indeed, the increase of light intensity by 150 *μ*mol m^−2^ s^−1^ could reduce the duration of the inductive phase from 60 to 40 days and it could slightly shorten the finishing time.

It is known that natural daily fluctuation of light intensity causes unpredictable results of the inductive treatment, but it is still unclear how constant supplemental lighting enhances the effect of cooling [29]. Some authors hypothesized that the promoting effect of extra lighting on flowering could be related to the increase in the availability of assimilates (as a consequence of the higher photosynthetic rate). Even considering the complexity of the light influence on CAM metabolism in* Phalaenopsis* [30], consistent with this hypothesis, in our experiment supplemental lighting increased the dry matter accumulation per plant.

Commercial quality improved when plants were induced under supplemental light, because the second flower stem and stem branching are highly appreciated by consumers in potted orchids. It is known that* Phalaenopsis* plants form at least two undifferentiated bud* primordia* that partially develop and then become dormant and that, under appropriate environmental conditions, the upper bud develops into a flower stem [31]; however no data seems to be available on the effect of light intensity on this process.

## 5. Conclusions

Our experiments provide useful information to optimize the cultivation protocol of* Phalaenopsis* orchids in Mediterranean greenhouses.

The exposure to inductive stimuli of plants 2 leaves smaller (approximately 3 months younger) than the optimal size negatively affected the flowering time and flower stem characteristics in “Sogo Yukidian” (Large), “Chain Xen Diamond” (Medium), and “Pinlong” (Small); however results revealed different behaviour in the hybrids. Therefore, from a commercial point of view, the convenience to anticipate flower induction should be evaluated by comparing the economic impact of these effects in the specific genotype to the reduction of production cost that could be achieved by shortening the vegetative phase.

Flower induction of* Phalaenopsis* “Premium” can be performed at 19/17°C instead of 21/19°C, without affecting flowering earliness and cut stem quality and improving the architecture of potted plants, with significant energy savings for greenhouse heating. In the same hybrid, increasing the light intensity during the 60-day phase of flower induction would allow reducing the treatment duration, to slightly anticipate flowering and to strongly improve the potted plants characteristics. In operative terms, higher radiation in Mediterranean regions could be easily achieved by reducing the rate of roof shading in the greenhouse compartment.

In conclusion, adjustments of the protocols for plant management (threshold size for flowering) and inductive treatment (cooling temperatures, light intensity, and time of exposure) could reduce the cost and raise the economical benefits of greenhouse production of* Phalaenopsis*. For this aim, considering the specific sensitivity of the hybrids to the proposed changes of the reference parameters would allow reducing the side effects on commercial quality, also depending on the purpose of cultivation (cut stems, potted plants) and the market requirements.

## Figures and Tables

**Table 1:** Normal plant and flower stem characteristics and optimal size for flowering in the 3 *Phalaenopsis* hybrids used in Experiment 1 (effects of plant size).

	Large (“Sogo Yukidian V3”)	Medium (“Chain Xen Diamond”)	Small (“Pinlong Cheris”)
Plant height (cm)	70	50	30
Flower width (cm)	Large (12)	Medium (9)	Small (5.5)
Flower colour	White	Mottled white	Pink
Optimal size (leaves/plant)	5	7	5
Production purpose	Cut stem/potted blooming plant	Potted blooming plant	Potted blooming plant

**Table 2:** Effects of the different treatments during the phase of flower induction on flowering time of *Phalaenopsis* hybrids. Within each factor, ns or different letters indicate, respectively, nonsignificant and significant differences at *P* = 0.05. Experiment 1 (effect of plant size): Large = “Sogo Yukidian”; Medium = “Chain Xen Diamond”; Small = “Pinlong”; *S*_opt_ = 5 leaves per plant in Large and Small hybrids and 7 leaves per plant in the Medium hybrid; *S*_red_ = 3 and 5 leaves per plant, respectively.  Experiment 2 (effects of thermal regime): hybrid “Premium”; *T*_ref_ = 21/19°C, *T*_low_ = 19/17°C.  Experiment 3 (effects of light intensity): hybrid “Premium”; *L*_ref_ = reference light regime; *L*_sup⁡_ = supplemental lighting with additional PPFD of 150 *μ*mol m^−2^ s^−1^ for 6 hours per day.

	Stem emergence (DBT)	First flower anthesis (DBT)	Complete anthesis of the inflorescence^*^ (DBT)
Experiment 1 (effect of the hybrid)			
Large	34.4^b^	112.5^b^	—
Medium	30.2^c^	106.6^c^	—
Small	46.4^a^	122.7^a^	—
Experiment 1 (effect of the plant size)			
*S*_opt_	34.5^b^	111.4^b^	—
*S*_red_	39.5^a^	116.5^a^	—

Experiment 2 (effects of thermal regime)			
*T*_ref_	47.5	160.6	196.5
*T*_low_	55.0	168.5	201.5
	ns	ns	ns

Experiment 3 (effects of light intensity)			
*L*_ref_	43.4^a^	149.3^a^	185.4
*L*_sup⁡_	35.9^b^	142.9^b^	180.2
			ns

^*^Time for complete anthesis is not determined in hybrids for potted blooming plant.

**Table 3:** Effects of the different treatments during the phase of flower induction on the main flower stem characteristics in *Phalaenopsis* hybrids. Within each factor, ns or different letters indicate, respectively, nonsignificant and significant differences at *P* = 0.05. Experiment 1 (effect of plant size): Large = “Sogo Yukidian”; Medium = “Chain Xen Diamond”; Small = “Pinlong”; *S*_opt_ = 5 leaves per plant in Large and Small hybrids and 7 leaves per plant in the Medium hybrid; *S*_red_ = 3 and 5 leaves per plant, respectively.  Experiment 2 (effects of thermal regime): hybrid “Premium”; *T*_ref_ = 21/19°C, *T*_low_ = 19/17°C.  Experiment 3 (effects of light intensity): hybrid “Premium”; *L*_ref_ = reference light regime, *L*_sup⁡_ = supplemental lighting with additional PPFD of 150 *μ*mol m^−2^ s^−1^ for 6 hours per day.

	Stem length (cm)	Stalk diameter (mm)	Inflorescence length (cm)	Number of flowers (*n*/stem)
Experiment 1 (effect of the hybrid)				
Large	61.8^a^	4.82^a^	21.3^a^	9.0 a
Medium	50.4^b^	4.79^a^	21.3^a^	9.6 a
Small	31.9^c^	3.01^b^	16.7^b^	13.0 b
Experiment 1 (effect of the plant size)				
*S*_opt_	51.4^a^	4.37^a^	22.2^a^	11.6 a
*S*_red_	44.7^b^	4.04^b^	17.3^b^	9.4 b

Experiment 2 (effects of thermal regime)				
*T*_ref_	65.6^a^	5.87	27.3^a^	10.14 a
*T*_low_	58.6^b^	6.18	22.0^b^	8.71 b
		ns		

Experiment 3 (effects of light intensity)				
*L*_ref_	58.1	5.98	21.5	7.9
*L*_sup⁡_	61.8	5.96	22.5	8.6
	ns	ns	ns	ns

## Abbreviations

CAM: Crassulacean acid metabolism
D/N: Day/night
DBT: Days from the beginning of the inductive thermal treatment
EC: Electrical conductivity
$L_{\text{ref}}$: Reference light regime
$L_{\sup}$: Supplemental lighting
PPFD: Photosynthetic photon flux density
R.H.: Relative humidity
$S_{\text{opt}}$: Optimal plant size
$S_{\text{red}}$: Reduced plant size
$T_{\text{low}}$: Moderately lower thermal regime
$T_{\text{ref}}$: Reference thermal regime.
